# Distinct osteoclast transcriptional states and heterotypic fusion arising from circulating pre-dendritic cells and monocytes

**DOI:** 10.3389/fcell.2026.1954531

**Published:** 2026-09-30

**Authors:** Patricia Riedlova, Sabarinadh Chilaka, Shatakshi Sood, John Cole, Cecilia Ansalone, Carl S. Goodyear

**Affiliations:** School of Infection and Immunity, University of Glasgow, Glasgow, United Kingdom

**Keywords:** osteoclasts, osteoclast fusion, heterotypic fusion, circulating precursors, monocyte precursors, dendritic cell precursors, transcriptional states

## Abstract

Osteoclasts (OCs) are multinucleated bone-resorbing cells essential for bone remodelling and contribute to pathological bone loss in inflammatory and metabolic bone diseases. Although classically considered to arise from monocyte/macrophage precursors, circulating pre-dendritic cells (pre-DCs) also retain osteoclastogenic potential. However, whether OCs derived from these distinct precursors retain different transcriptional states, and how they interact during fusion, remain unclear. Here, we compared OCs generated from directly isolated circulating monocytes and pre-DCs and investigated how these populations contribute to OC formation and fusion within mixed cultures. RNA sequencing identified 41 genes significantly enriched in pre-DC-derived OCs and 9 in monocyte-derived OCs, while core osteoclastogenic and fusion-associated genes showed no significant differential expression. Gene set enrichment analysis (GSEA) identified distinct precursor-associated transcriptional programmes. Pre-DC-derived OCs were enriched for extracellular matrix organisation, skeletal development, canonical Wnt signalling and immune-regulatory pathways, whereas monocyte-derived OCs were enriched for cell-cycle, proteasomal, lysosomal and innate immune pathways. In mixed cultures, OC output increased with the proportion of pre-DCs, with the greatest OC formation observed in pre-DC-only cultures. Live-cell imaging demonstrated heterotypic fusion, with monocyte-derived precursors observed fusing with established pre-DC-derived multinucleated OCs, whereas exclusively monocyte-derived multinucleated OCs were infrequently observed. These findings indicate that both populations generate OCs with a conserved core transcriptional profile but distinct precursor-associated transcriptional states. Greater pre-DC abundance was associated with increased OC output, and live-cell imaging showed that monocyte-derived cells can be incorporated into pre-DC-derived multinucleated OCs. This heterogeneity may influence OC abundance, multinucleation, and functional properties in inflammatory and metabolic bone diseases.

## Introduction

Osteoclasts (OCs) are multinucleated bone-resorbing cells that play a central role in skeletal remodelling. Although they are most commonly considered to arise from monocyte/macrophage precursors, several myeloid populations can enter the OC lineage, including dendritic-lineage precursors (pre-DCs) (Tsai et al., 2023; Puchner et al., 2024). Evidence supporting a dendritic cell (DC) contribution to osteoclastogenesis has emerged from both murine and human studies, although the precursor populations examined have differed. Murine depletion, fate-mapping and differentiation studies have demonstrated that *bona fide* dendritic-lineage populations can contribute to OC formation (Puchner et al., 2024). In contrast, earlier human transcriptomic and functional comparisons used DCs generated *ex vivo* from blood monocytes through GM-CSF and IL-4 stimulation rather than pre-DCs isolated directly from peripheral blood (Gallois et al., 2010; Narisawa et al., 2021). We previously showed that directly isolated circulating CD14^−^CD16^−^CD11c^+^ pre-DCs and CD14^+^ monocytes represent distinct human OC precursor populations with different responses to osteoclastogenic and inflammatory signals (Ansalone et al., 2021). Whether OCs derived from these distinct circulating precursors converge completely on a common mature phenotype or retain precursor-associated transcriptional states remains unknown.

OC formation also requires coordinated cell-cell fusion, yet the cellular organisation of this process remains incompletely understood (Ishii and Saeki, 2008; Hobolt-Pedersen et al., 2014). M-CSF and RANK–RANKL signalling establish the osteoclastogenic programme through transcription factors including NFATc1, while DC-specific transmembrane protein (DC-STAMP) and OC-stimulatory transmembrane protein (OC-STAMP) are key regulators of multinucleation (Jiang et al., 2023; Yagi et al., 2005; Homma et al., 2025; Zhu et al., 2026). Murine studies established the essential roles of DC-STAMP and OC-STAMP and identified heterogeneous DC-STAMP^low^ and DC-STAMP^high^ precursor populations, with DC-STAMP^low^ cells acting as master fusogens and DC-STAMP^high^ cells contributing as mononuclear fusion partners (Yagi et al., 2005; Homma et al., 2025; Mensah et al., 2010). Complementary studies using human monocyte-derived OCs showed that fusion frequently occurs between cells differing in differentiation state, mobility and nuclearity (Hobolt-Pedersen et al., 2014; Søe et al., 2015). However, whether distinct circulating human precursor lineages, such as pre-DCs and monocytes, make complementary contributions during OC formation remains unclear.

This question may be particularly relevant to inflammatory and metabolic bone diseases, in which the composition and activation state of the myeloid precursor pool, together with the availability of osteoclastogenic signals, can be altered (Tsai et al., 2023; Sun et al., 2021). Distinct precursor populations may therefore influence not only the number of OCs formed, but also their transcriptional state, multinucleation and functional behaviour. In this study, we compared the transcriptional profiles of OCs generated from directly isolated human pre-DCs and monocytes and investigated how these precursor populations contribute to OC formation and fusion within mixed cultures.

## Methods

### Isolation of CD14^+^ monocytes and pre-dendritic cell precursors from peripheral blood

Peripheral blood from healthy donors was collected in EDTA vacuum blood collection tubes (BD Vacutainer). The study protocol was approved by the West of Scotland Research Ethics Committee (11/S0704/7), and all donors provided written informed consent. Alternatively, buffy coats were obtained from the Scottish National Blood Transfusion Service or from Newcastle blood services. Peripheral blood mononuclear cells (PBMCs) were isolated by density gradient centrifugation using Ficoll-Paque PLUS (GE Healthcare Life Science). CD14^+^ monocytes and myeloid pre-DCs were magnetically enriched from PBMCs using the EasySep Human CD14 Positive Selection Kit and EasySep Human Myeloid DC Enrichment Kit (STEMCELL Technologies), respectively. Cell purity was assessed by flow cytometry and was consistently ≥96%. Following isolation, cells were resuspended at 1 × 10^6^ cells/mL in complete α-MEM medium supplemented with 10% heat-inactivated foetal bovine serum (FBS), 0.02 mM L-glutamine, 10 units/mL penicillin, and 0.1 mg/mL streptomycin (Invitrogen, Thermo Fisher Scientific) and used for downstream applications.

### Osteoclast differentiation and culture

Isolated CD14^+^ monocytes and pre-DCs were cultured overnight in complete α-MEM supplemented with macrophage colony-stimulating factor (M-CSF, 25 ng/mL; PeproTech). Osteoclast differentiation was induced by addition of recombinant human RANKL (50 ng/mL; PeproTech). For mixed precursor experiments, CD14^+^ monocytes and pre-DCs were plated together at defined ratios and cultured for 120 h following RANK-L addition, with medium replacement on day 3. Cells were fixed and stained for tartrate-resistant acid phosphatase (TRAP; Sigma-Aldrich), imaged using an EVOS imaging system, and osteoclast numbers quantified using ImageJ. For cell sorting experiments, CD14^+^ monocytes and pre-DCs were differentiated separately under the same conditions and harvested at day 6 for sorting.

### Cell sorting of mature osteoclasts and RNAseq

CD14^+^ monocytes were differentiated into osteoclasts (OCs) in the presence of M-CSF and RANKL, for 5 days (∼120 h) from RANKL addition as described above. Cells were washed with warm PBS and detached using Accutase (37 °C, 20 min), collected, centrifuged (300 × g, 5 min), resuspended in PBS, and counted by trypan blue. Cells were adjusted to 1 × 10^6^ cells/mL. An aliquot (1 × 10^5^ cells) was reserved as an unstained control. The remaining cells were stained with a live/dead viability dye (1:750, 10 min, room temperature, protected from light) in serum-free conditions. Cells were then washed and resuspended in sorting buffer (PBS without Ca^2+^/Mg^2+^, 1% FBS, 5 mM EDTA). For flow cytometric analysis, cells were stained with fluorophore-conjugated antibodies against CD14 and OSCAR, with appropriate isotype controls, for 30 min at 4 °C in the dark. After washing, cells were resuspended in cold sorting buffer, filtered through a 70 μm mesh, and subjected to cell sorting using a BD FACSAria™ III cell sorter (BD Biosciences) equipped with a 100 µm nozzle. Osteoclasts and precursors were defined as CD14^−^OSCAR^+^ cells, with gating established based on isotype controls. Sorted cells were collected directly into RLT lysis buffer (Qiagen) supplemented with β-mercaptoethanol and immediately snap-frozen for downstream RNA extraction. Typical yields were approximately 1 × 10^5^ sorted cells from ∼1 × 10^7^ input cells.

### RNA extraction, library preparation and sequencing

Total RNA was extracted from sorted pre-DC-derived and monocyte-derived osteoclasts (OCs) using the RNeasy Micro Kit (Qiagen), according to the manufacturer’s instructions. Total RNA (500 ng) was subjected to ribosomal RNA depletion using the NEBNext rRNA Depletion Kit (Human/Mouse/Rat) (New England Biolabs, #E6310) according to the manufacturer’s instructions. Directional RNA-seq libraries were constructed using the NEBNext Ultra II Directional RNA Library Prep Kit for Illumina (New England Biolabs, #E7760). Indexed libraries were pooled and sequenced on an Illumina NextSeq 500 platform to generate approximately 50 million paired-end reads (2 × 75 bp) per sample.

### RNAseq analysis

RNAseq data was analysed using R and visualised with RStudio (version 4.5.0). Raw read counts were normalised using the DESeq2 workflow, and differential expression analysis between pre-DC-derived and monocyte-derived osteoclasts (n = 4 per precursor population) was assessed using the Wald test implemented in DESeq2. P-values were adjusted for multiple testing using the Benjamini–Hochberg method. Genes with an adjusted p-value (p.adj <0.01) and an absolute log2 fold change> 1.5 were considered significantly differentially expressed. Further plots used for visualisation of the data (volcano plots, heatmap, etc.) were computed using ggplot2, dplyr, ggforce, reshape/reshape2, amap, and base R. Gene set enrichment analysis (GSEA) was performed using the clusterProfiler R package on ranked gene lists based on log2 fold change values, using the gseGO function with Gene Ontology (biological process (BP), molecular function (MF) and cellular component (CC)) annotations (via org.Hs.eg.db), and results were visualised using ggplot2 and enrichplot.

### Live cell imaging

For live-cell imaging experiments, CD14^+^ monocytes and pre-DCs were labelled right after isolation using CellTracker Green CMFDA and CellTracker Orange CMRA (Thermo Fisher Scientific). Dyes were reconstituted in anhydrous dimethyl sulfoxide (Sigma-Aldrich) and used at a final concentration of 5 µM in serum-free αMEM for 30 min at 37 °C. Cells were washed and returned to complete osteoclast culture medium containing 25 ng/mL M-CSF. To account for differences in differentiation kinetics between precursor populations, cells were cultured under differential RANKL concentrations while maintaining identical culture duration. CD14^+^ monocytes were stimulated with 50 ng/mL RANKL, whereas pre-DCs were stimulated with 1 ng/mL RANKL. Cells were cultured for 96 h to generate mononuclear or small multinucleated (2–3 nuclei) pre-osteoclasts. Following differentiation into pre-osteoclasts, labelled cells were harvested using 0.05% Trypsin/EDTA, mixed at a 1:1 ratio, and seeded onto 18-well glass chamber slides (Ibidi) at 100,000 total cells per well in osteoclast differentiation medium containing M-CSF and RANKL. Cells were allowed to adhere and differentiate further for approximately 24 h prior to imaging. Live-cell imaging was performed on a Leica DMi8 inverted fluorescence microscope (Leica Microsystems). Images were acquired sequentially at each time point in the following order: brightfield, green fluorescence, and orange fluorescence to minimise spectral bleed-through. Time-lapse images were acquired at approximately 10-min intervals overnight to monitor cell movements, interactions, and fusion events during osteoclast differentiation.

## Results

To compare osteoclastogenesis from distinct precursor populations, pre-DCs and monocytes were differentiated into OCs under osteoclastogenic conditions, and the resulting OCs were analysed by RNA-sequencing (RNASeq). Principal component analysis (PCA) demonstrated that separation according to precursor origin was most apparent along PC2 (21.83% of the total variance), while PC1 (32.69%) captured greater overall variation but did not consistently distinguish the two populations (Figure 1A). Differential expression analysis identified 41 genes significantly upregulated in pre-DC-derived OCs, compared with 9 genes upregulated in monocyte-derived OCs (Figure 1B). Hierarchical clustering further demonstrated distinct expression patterns between the two populations (Figure 1C).

**FIGURE 1:**
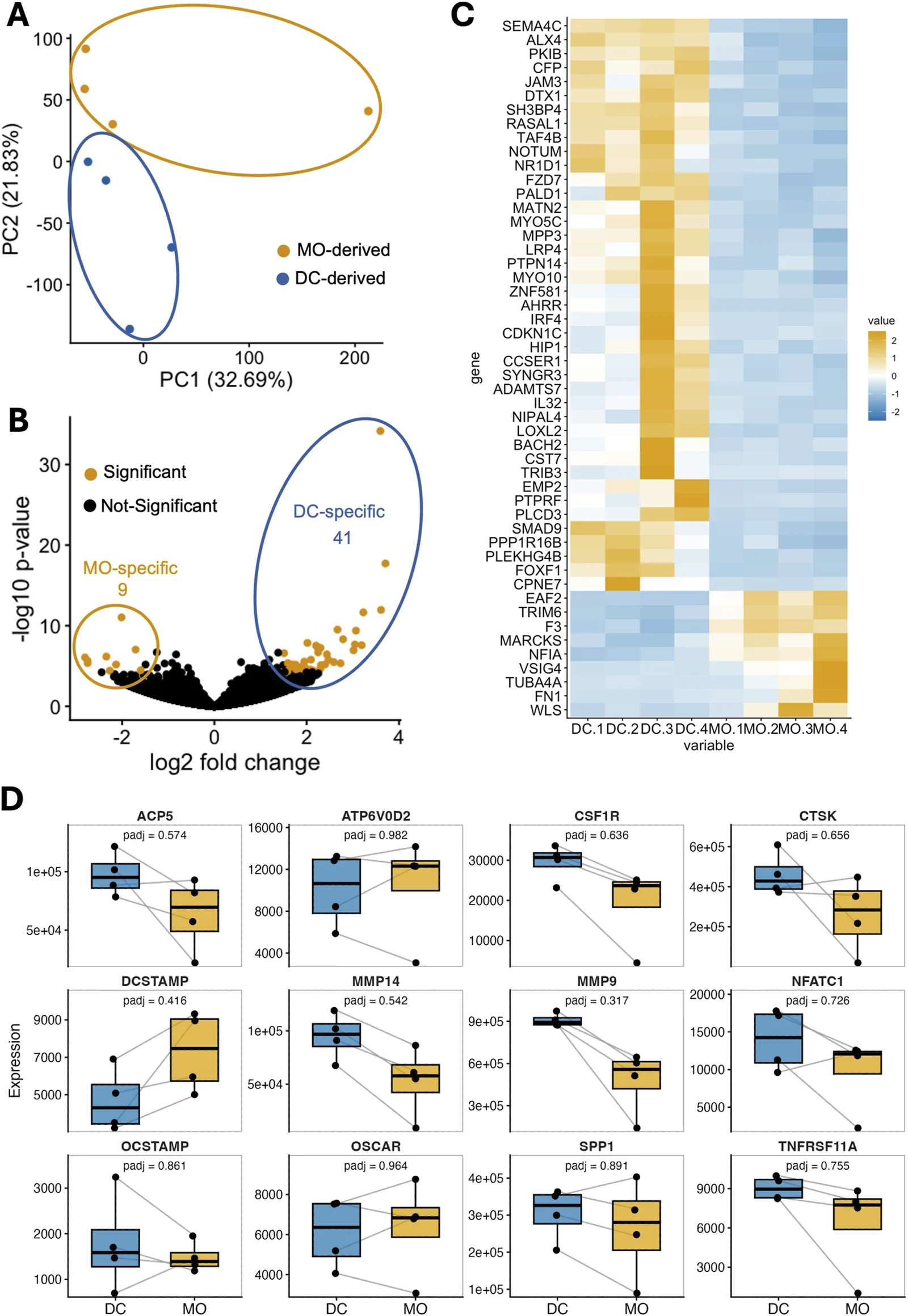
RNAseq data analysis of dendritic cell precursor- and monocyte-derived osteoclasts. **(A)** PCA plot of dendritic cell precursor (pre-DC)- and monocyte (MO)- derived osteoclasts (OCs) showing separation along PC1 (32.69%) and PC2 (21.83%). **(B)** Volcano plot showing differentially expressed genes between pre-DC-derived (blue ellipse) and MO-derived (yellow ellipse) OCs. Significantly differentially expressed genes are shown in yellow, and group-specific genes are indicated with a corresponding gene count. Non-significant genes are shown in black. **(C)** Heatmap of significantly differentially expressed genes across individual samples, with hierarchical clustering and Z-score-scaled expression. **(D)** Boxplots of selected OC-associated genes in pre-DC-derived and monocyte-derived OCs, with paired donor samples connected by grey lines and adjusted p-values shown.

To determine whether both populations retained a shared OC transcriptional identity, we examined the expression of key osteoclastogenic genes, including tartrate-resistant acid phosphatase (*ACP5*), ATPase H+ transporting V0 subunit d2 (*ATP6V0D2*), CSF1 receptor (*CSF1R*), *CTSK*, *DCSTAMP*, *MMP9*, *MMP14*, *NFATC1*, *OCSTAMP*, OC-associated receptor (*OSCAR*), secreted phosphoprotein 1 (*SPP1*), and tumour necrosis factor receptor superfamily member 11A (*TNFRSF11A*), in pre-DC- and monocyte-derived OCs. Notably, there was no significant differential expression between the two OC populations following multiple-testing correction. However, paired donor analysis suggested higher expression of *MMP9 and MMP14* in pre-DC-derived OCs, whereas *DCSTAMP* expression was generally higher in monocyte-derived OCs, showing a donor-matched directional trend across samples. The remaining genes showed broadly comparable expression between the two populations (Figure 1D). These findings indicate that OCs derived from pre-DCs and monocytes share a conserved core OC transcriptional programme despite broader precursor-associated differences.

To characterise these broader transcriptional differences, gene set enrichment analysis (GSEA) was performed using the combined Gene Ontology (GO) biological process, molecular function and cellular component collections. Selected significant pathways representing the principal biological processes are shown in Figure 2, with the complete analysis, and core-enrichment genes for the displayed pathways, provided in Supplementary Material S1. Pathways with positive normalised enrichment scores (NES) were enriched in pre-DC-derived OCs and included collagen fibril organisation, extracellular matrix organisation, bone and skeletal system development, canonical Wnt signalling, cytokine receptor activity, and lymphocyte differentiation (Figure 2; Supplementary Material S1). In contrast, pathways with negative NES values were enriched in monocyte-derived OCs and included lipid catabolic processes, mitochondrial translation, response to bacterium, primary lysosome, mitotic sister chromatid segregation, and proteasome complex pathways (Figure 2; Supplementary Material S1). Thus, precursor origin is associated with distinct transcriptional states in mature OCs despite conservation of their core OC identity.

**FIGURE 2:**
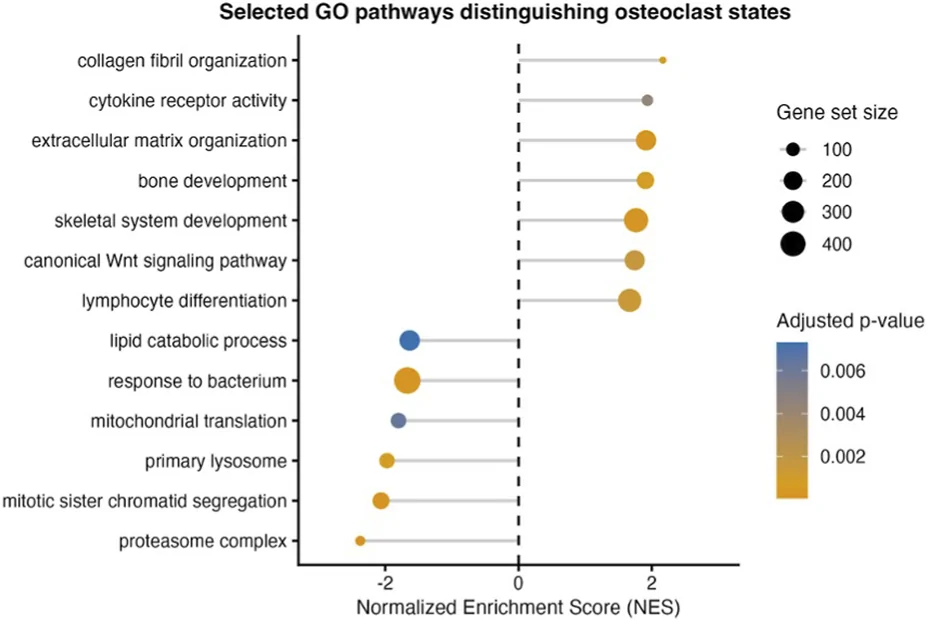
Selected Gene Ontologies pathways distinguishing osteoclast derived from monocytes and dendritic cell precursors. Gene set enrichment analysis (GSEA) across all Gene Ontologies (GO; biological process, molecular function, and cellular component). Normalised enrichment scores (NES) indicate pathways enrichment in dendritic cell precursor-derived osteoclasts (positive NES) or monocyte-derived osteoclasts (negative NES). Individual points show gene set size involved in each pathway, and colour corresponds to adjusted p-value. The dashed vertical line indicates NES = 0.

We next examined whether precursor composition influenced OC formation within mixed cultures containing donor-matched pre-DCs and monocytes. Pre-DC-only cultures generated the greatest number of mature OCs, while progressively fewer mature OCs were observed as the proportion of monocytes increased (Figure 3A). Quantification of TRAP-positive multinucleated OCs confirmed this graded pattern, with the highest OC numbers in pre-DC-only cultures and decreasing numbers across cultures containing increasing proportions of monocytes (Figure 3B). Similarly, the proportion of the culture area occupied by TRAP-positive OCs decreased progressively as the proportion of monocytes increased (Figure 3C). These findings demonstrate a positive association between pre-DC representation and total OC output within mixed precursor cultures.

**FIGURE 3:**
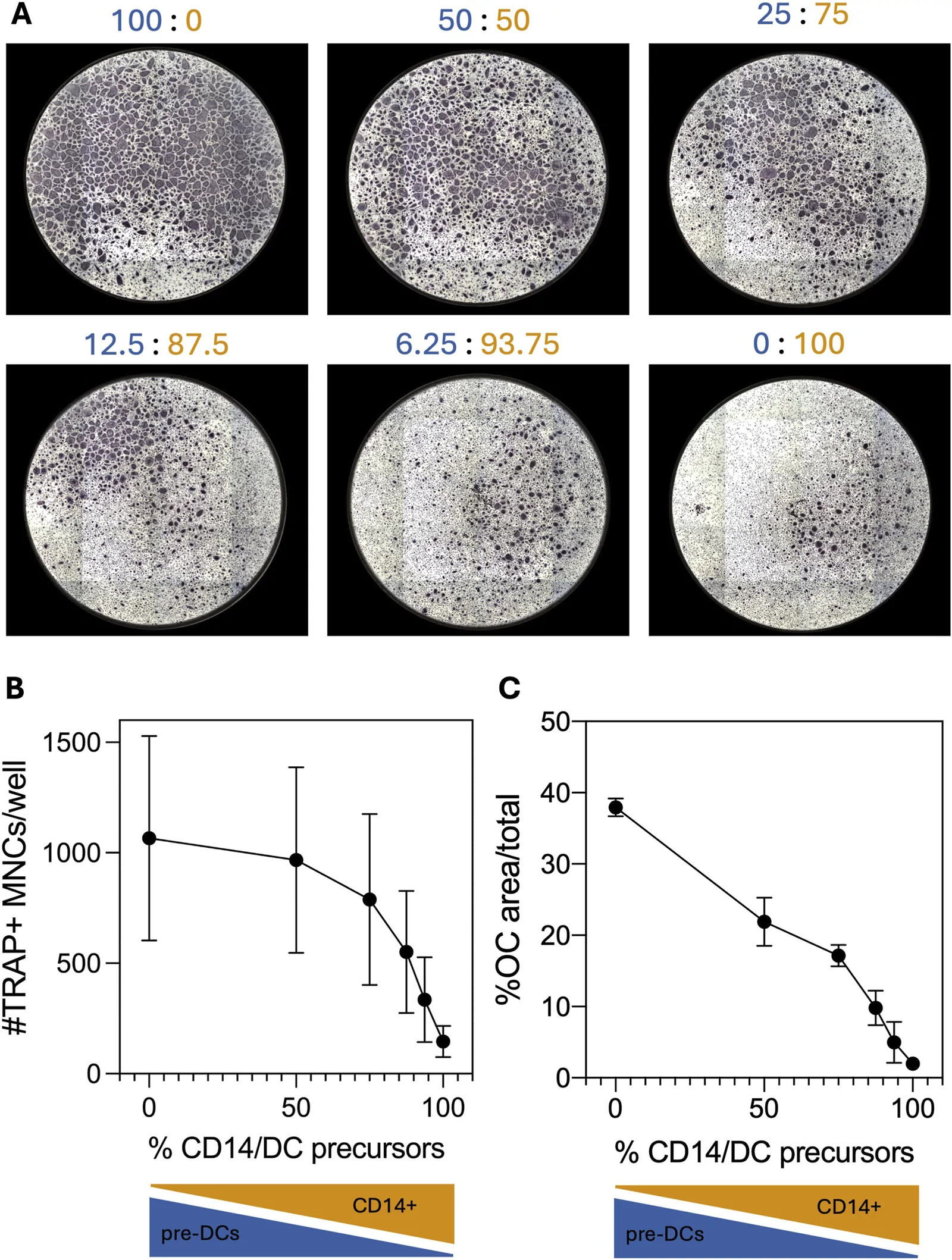
Osteoclast differentiation in mixed co-cultures of dendritic cell precursors and monocytes. **(A)** Representative TRAP-stained images of OC formation under the indicated culture conditions. The ratios shown above each image indicate the starting proportions, expressed in percentages, of dendritic cell precursors (pre-DCs; blue) and CD14^+^ monocytes (yellow) within each culture condition. TRAP-positive osteoclasts (OCs) are visualised in dark purple. **(B)** Quantification of TRAP-positive multinucleated OCs (MNCs) across cultures containing different proportions of autologous pre-DCs and monocytes (CD14^+^). Error bars show mean ± SD of n = 3 individual donors. **(C)** Representative quantification of the culture area occupied by TRAP-positive osteoclasts across the different precursor mixtures. Error bars show mean ± SD of technical replicates of one representative donor.

To investigate how the two precursor populations contributed to multinucleated OC formation, pre-DCs and monocytes were fluorescently labelled prior to co-culture. In representative static fluorescence images multinucleated OCs were predominantly derived from pre-DCs or contained both pre-DC- and monocyte-derived labels, whereas exclusively monocyte-derived multinucleated OCs were infrequently observed (Figure 4A; Supplementary Material S2). Time-lapse live-cell imaging subsequently captured monocyte-derived pre-OCs fusing with established pre-DC-derived multinucleated OCs (Figure 4B). Fusion between monocyte-derived cells resulting in exclusively monocyte-derived multinucleated OCs was not evident in the representative sequences analysed. Together, these observations demonstrate that cells arising from the two precursor lineages can participate in heterotypic OC fusion and are consistent with complementary contributions to multinucleation.

**FIGURE 4:**
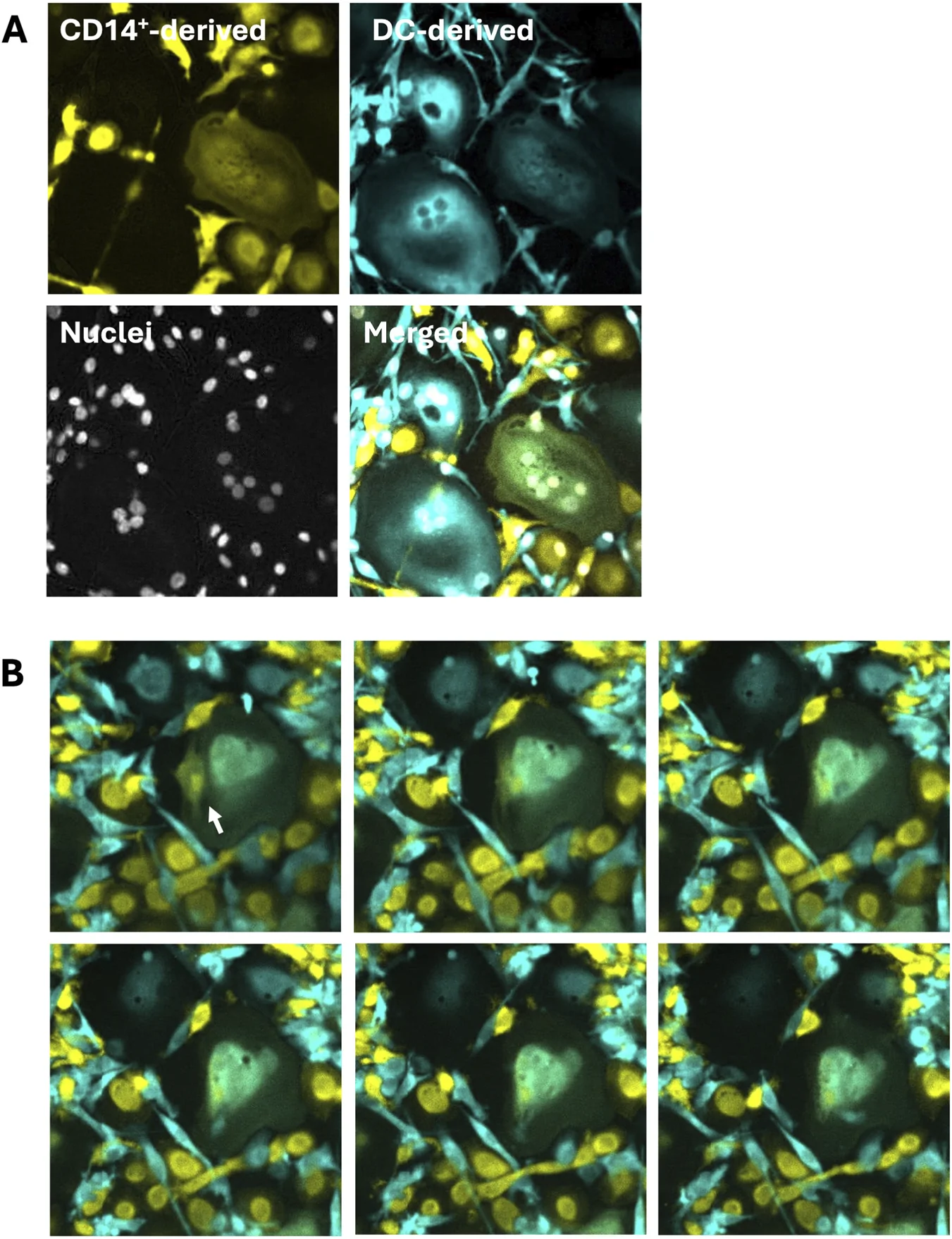
Live-cell imaging of osteoclast fusion in mixed dendritic cell precursor and monocyte co-cultures. **(A)** Representative live-cell fluorescence images showing CD14^+^ monocyte-derived cells and osteoclasts (yellow), dendritic cell precursor-derived cells and osteoclasts (cyan), nuclei (grey), and the corresponding merged image. **(B)** Representative time-lapse sequence showing a fusion event between a monocyte-derived precursor and a multinucleated pre-DC-derived osteoclast. The site of the fusion event is indicated by a white arrow. Images are representative of three independent experiments.

## Discussion

The principal finding of this study is that directly isolated circulating pre-DCs and monocytes generate mature OCs that converge on a conserved core OC transcriptional programme while retaining distinct precursor-associated transcriptional states. Both populations expressed comparable levels of established genes involved in OC differentiation, fusion and resorptive function, but differed in their broader transcriptional profiles and pathway enrichment. In mixed cultures, increasing pre-DC representation was associated with greater OC formation, while lineage-labelled imaging showed that monocyte-derived cells could fuse with established pre-DC-derived multinucleated OCs. Together, these findings identify precursor origin as a contributor to mature OC state and demonstrate that distinct circulating human precursor populations can cooperate during heterotypic OC fusion.

The shared expression of core OC-associated genes indicates that both precursor populations acquire a recognisable OC identity following M-CSF and RANKL stimulation, consistent with the differentiation programme established through RANKL–RANK signalling and NFATc1 activation (Jiang et al., 2023; Zhu et al., 2026). At the same time, separation by PCA, hierarchical clustering and differential expression analysis demonstrates that osteoclastogenic differentiation does not completely erase features associated with precursor origin. This extends our previous observation that epigenetically distinct circulating human OC precursors respond differently to osteoclastogenic and inflammatory signals (Ansalone et al., 2021). Importantly, earlier human transcriptomic comparisons used DCs generated *ex vivo* from blood monocytes through GM-CSF and IL-4 stimulation, whereas the present study examined pre-DCs isolated directly from peripheral blood before osteoclastogenic differentiation (Gallois et al., 2010; Narisawa et al., 2021). Our findings therefore show that phenotypically distinct circulating human precursors can generate mature OCs with a shared lineage identity but distinguishable transcriptional states.

These precursor-associated states may contribute to functional OC heterogeneity. Directional differences were observed in several genes linked to OC activity, including *MMP9, ACP5, CTSK, MMP14, and DCSTAMP.* Although these differences did not reach statistical significance after multiple-testing correction, they showed a consistent donor-matched trend across samples. These patterns are notable in light of previous studies showing enhanced osteoclastogenic and bone-resorptive capacity in DC-derived OCs compared with monocyte-derived OCs (Gallois et al., 2010; Narisawa et al., 2021). Murine and human studies have also supported the ability of *bona fide* dendritic-lineage populations to contribute efficiently to OC formation (Puchner et al., 2024). The transcriptional differences identified here may therefore reflect functional diversity that extends beyond acquisition of a common core OC identity.

Pathway analysis provided further evidence that the two populations reach distinct mature OC states. Pre-DC-derived OCs were enriched for programmes associated with extracellular matrix organisation, skeletal development, canonical Wnt signalling and immune regulation. In contrast, monocyte-derived OCs showed enrichment of lipid catabolism, mitochondrial translation, lysosomal and proteasomal functions, cell-cycle regulation and innate immune responses. These findings indicate precursor-associated differences in pathways linked to extracellular matrix organisation, metabolism and immune regulation. Rather than representing a uniform terminal population, human OCs may therefore comprise multiple transcriptional states shaped partly by the developmental and regulatory properties of their precursors, consistent with evidence of extensive transcriptional reprogramming and heterogeneity during human osteoclastogenesis (Hansen et al., 2024).

Among the genes enriched in pre-DC-derived OCs, IRF4 is of interest given its previously reported roles in osteoclastogenesis and myeloid multinucleation. Specifically, IRF4 has previously been implicated in RANKL-induced osteoclastogenesis through regulation of NFATc1 (Nakashima and Haneji, 2013). More recently, a preprint by Hofmann et al. identified IRF4 as a central regulator of fusion competence during multinucleated giant-cell formation, where it controlled DC-STAMP, OC-STAMP and broader cell-fusion programmes (Hofmann et al., 2025). In that model, IRF4 defined an alternative programme of myeloid multinucleation distinct from canonical RANKL-driven osteoclastogenesis. In the present dataset, IRF4 was enriched in pre-DC-derived OCs and reached statistical significance in the differential expression analysis. Although inter-donor variability in absolute expression levels was observed, the directionality of pre-DC enrichment was preserved across the donors analysed, supporting an association with the pre-DC-derived OC state. Its enrichment in pre-DC-derived OCs may therefore reflect retention of a precursor-associated or giant-cell-like regulatory programme that contributes to the distinct state of these cells. Enrichment of Wnt-associated genes further suggests differential engagement of pathways involved in skeletal development and bone remodelling as candidate features of the pre-DC-derived OC state. Together, these observations point to IRF4 and Wnt-related pathways as supported and biologically plausible contributors to the distinct transcriptional state observed in pre-DC-derived OCs, although further studies will be required to define their functional role.

The mixed-culture experiments provided functional context for the transcriptional findings. OC number and area were greatest in pre-DC-only cultures and decreased progressively as the proportion of monocytes increased, demonstrating that the composition of the precursor pool can shape total OC output. This graded relationship is consistent with previous reports that dendritic-lineage cells can generate OCs efficiently and can produce large, highly multinucleated and strongly resorptive cells (Puchner et al., 2024; Gallois et al., 2010; Narisawa et al., 2021). These findings reinforce the concept that directly isolated pre-DCs are not simply an alternative source of OCs, but a precursor population with particularly strong osteoclastogenic capacity.

Lineage-labelled imaging further showed that the two precursor populations can contribute to the same multinucleated OC. Monocyte-derived cells were observed fusing with established pre-DC-derived multinucleated OCs, while exclusively monocyte-derived multinucleated OCs were infrequently observed in the representative cultures. This pattern is consistent with human imaging studies showing that OC fusion occurs between partners differing in differentiation state, mobility and nuclearity, with motile mononuclear cells sequentially contributing nuclei to established multinucleated OCs (Hobolt-Pedersen et al., 2014; Søe et al., 2015). Our observations extend this model by showing that the established multinucleated OC and the incoming mononuclear fusion partner can arise from distinct circulating precursor lineages. Pre-DCs and monocytes may therefore generate transcriptionally distinct OC states while also cooperating during the formation of lineage-mixed multinucleated cells. However, quantification of nuclear class distribution and lineage-defined fusion events will be required to determine the relative contribution of each precursor population to OC multinucleation.

DC-STAMP provides a plausible mechanistic framework for this cooperation. Previous studies have demonstrated that DC-STAMP and OC-STAMP are key regulators of OC fusion and have described functionally distinct DC-STAMP^low^ and DC-STAMP^high^ precursor populations in murine models, with DC-STAMP^low^cells acting as master fusogens and DC-STAMP^high^ cells contributing as mononuclear fusion partners (Yagi et al., 2005; Homma et al., 2025; Mensah et al., 2010). In the present dataset, DC-STAMP expression was numerically higher in monocyte-derived OCs than in pre-DC-derived OCs, a direction that is consistent with our imaging observations showing monocyte-derived cells fusing with established pre-DC-derived multinucleated OCs. Overall, the transcriptomic and imaging observations are consistent with pre-DCs and monocytes making complementary contributions to OC multinucleation. While the direction of DC-STAMP expression aligns with previous descriptions of DC-STAMP^low^ and DC-STAMP^high^ fusion partners, the difference was not statistically significant in our dataset. Further studies will therefore be needed to establish whether these precursor populations play distinct roles during OC fusion.

This model may have particular relevance to inflammatory and metabolic bone diseases, in which the abundance, activation state and recruitment of myeloid precursor populations are altered. Pathological osteoclastogenesis may depend not only on local M-CSF and RANKL availability, but also on which precursor populations are present and how they interact during differentiation and fusion. Changes in precursor composition could consequently influence OC abundance, transcriptional state, cellular composition and functional behaviour. Defining these lineage-associated OC states may therefore reveal clinically relevant forms of OC heterogeneity and identify new opportunities for targeting pathological bone remodelling.

Several considerations will guide the next stages of this work. The present study used peripheral blood cells from a limited number of healthy donors under defined *ex vivo* osteoclastogenic conditions, and extending these analyses to tissue-resident and disease-associated precursors will establish their relevance *in vivo*. Bulk RNA-seq captured robust population-level differences but could not resolve heterogeneity within each OC population or the transcriptional state of individual fusion partners. Direct resorption assays will be needed to determine whether the identified transcriptional states translate into differences in bone-resorptive activity. In addition, quantitative analysis of lineage-defined fusion events, including fusion index, nuclear class distribution, and direct measurement of DC-STAMP in individual fusion partners will be required to test whether pre-DC- and monocyte-derived cells play distinct roles during OC fusion. Single-cell profiling, lineage tracing and functional assays will therefore provide important complementary approaches for refining the model proposed here.

## Conclusion

In conclusion, directly isolated circulating pre-DCs and monocytes generate mature OCs with a conserved core OC transcriptional programme but distinct precursor-associated transcriptional states. These states may contribute to functional OC heterogeneity, as suggested by previous reports of differential osteoclastogenic and resorptive activity among dendritic-lineage- and monocyte-derived OCs. Lineage-labelled co-cultures further demonstrated that pre-DC- and monocyte-derived cells can participate in heterotypic OC fusion, supporting a model in which distinct circulating myeloid precursor populations cooperate during multinucleation. These findings highlight precursor origin as an underappreciated determinant of mature OC state, formation and cellular composition, while identifying candidate mechanisms that should be tested in future functional studies.

## Data Availability

The datasets presented in this study can be found in online repositories. The names of the repository/repositories and accession number(s) can be found below: https://www.ncbi.nlm.nih.gov/geo/, GSE341844.
